# Role of hyperpolarization-activated cyclic nucleotide-gated ion channels in neuropathic pain: a proof-of-concept study of ivabradine in patients with chronic peripheral neuropathic pain

**DOI:** 10.1097/PR9.0000000000000967

**Published:** 2021-10-18

**Authors:** Shannon A. Bernard Healey, Ingrid Scholtes, Mark Abrahams, Peter A. McNaughton, David K. Menon, Michael C. Lee

**Affiliations:** a Division of Anaesthesia, University of Cambridge, Cambridge, United Kingdom; b Pain Service, Cambridge University Hospitals NHS Trust, Cambridge, United Kingdom; c Wolfson Centre for Age-Related Diseases, King's College London, London, United Kingdom

**Keywords:** Clinical trial, Hyperpolarization-activated cyclic nucleotide-gated channels, Ivabradine, Chronic pain, Diabetic neuropathies

## Abstract

**Introduction:**

Hyperpolarization-activated cyclic nucleotide-gated (HCN) ion channels mediate repetitive action potential firing in the heart and nervous system. The HCN2 isoform is expressed in nociceptors, and preclinical studies suggest a critical role in neuropathic pain. Ivabradine is a nonselective HCN blocker currently available for prescription for cardiac indications. Mouse data suggest that ivabradine in high concentrations is equianalgesic with gabapentin. We sought to translate these findings to patients with chronic peripheral neuropathic pain.

**Objectives:**

We sought to translate these findings to patients with chronic peripheral neuropathic pain.

**Methods:**

We adopted an open-label design, administering increasing doses of ivabradine to target a heart rate of 50 to 60 BPM, up to a maximum of 7.5 mg twice daily. All participants scored their pain on an 11-point numerical rating scale (NRS).

**Results:**

Seven (7) participants received the drug and completed the study. There was no significant treatment effect on the primary endpoint, the difference between the mean score at baseline and at maximum dosing (mean reduction = 0.878, 95% CI = −2.07 to 0.31, *P* = 0.1). Exploratory analysis using linear mixed models, however, revealed a highly significant correlation between ivabradine dose and pain scores (χ^2^(1) = 74.6, *P* < 0.001), with a reduction of 0.12 ± 0.01 (SEM) NRS points per milligram. The 2 participants with painful diabetic neuropathy responded particularly well.

**Conclusion:**

This suggests that ivabradine may be efficacious at higher doses, particularly in patients with diabetic neuropathic pain. Importantly, participants reported no adverse effects. These data suggest that ivabradine, a peripherally restricted drug (devoid of central nervous system side effects), is well tolerated in patients with chronic neuropathic pain. Ivabradine is now off-patent, and its analgesic potential merits further investigation in clinical trials.

## Abstract 2

Hyperpolarization-activated cyclic nucleotide-gated (HCN) channel receptors mediate neuropathic pain in preclinical models. Here, exploratory analysis reveals a dose-dependent reduction in pain with HCN blockade in patients with neuropathic pain.

## 1. Introduction

Hyperpolarization-activated cyclic nucleotide-gated (HCN) ion channels are important modulators of action potential rhythm and frequency in the heart and nervous system.^18,35^ They carry an inward current, named I_f_ in the heart and I_h_ in neurons.^18,35^ The HCN ion channel family comprises 4 isoforms, HCN1 to HCN4. HCN1 and HCN3 are relatively insensitive to modulation by cyclic AMP (cAMP), whereas in HCN2 and HCN4, the binding of cAMP shifts the activation curve as a function of membrane voltage in the positive direction and so increases the I_h_ current.^18,35^ HCN4 is expressed in pacemaker tissue in the heart and is responsible for the cardiac pacemaker potential.^18^ HCN1 and HCN2 are expressed in the central and peripheral nervous systems, where they are have a similar role in driving repetitive firing in neurons.^10,15,23,24,28,29,35^ Small sensory neurons, the majority of which are nociceptors, express a slow, cAMP-sensitive I_h_ current, consistent with HCN2 expression.^6,10,22,35^ Upon tissue injury, inflammatory mediators such as prostaglandin E2 are released, which results in a rise in intracellular cAMP and an increase in the rate of action potential firing.^24^ This mechanism is I_h_-dependent and therefore suggests that HCN2 may have a role as a “pacemaker of pain.”^12,24^ Preclinical work in mouse models of both inflammatory and neuropathic pain supports this role for HCN2. Pharmacological blockade and targeted genetic deletion of HCN2 channels reduced both in vitro nociceptor excitability and in vivo behavioural measures of pain.^7,11,22^

Neuropathic pain is notoriously difficult to treat and is associated with significant morbidity and mortality. Preclinical work suggests that HCN2 may have a particularly important role in neuropathic pain.^6,9,11,17,19,31^ In a sciatic nerve lesion model, there was no enhanced sensitivity to heat, cold, or mechanical stimuli in mice with a selective deletion of HCN2 in nociceptors.^11^ In a chemotherapy-induced neuropathy model, selective blockade of HCN1/HCN2 reduced neuronal excitability in vitro and thermal hypersensitivity in vivo*.*^9,31^ Although there is currently no HCN2-specific antagonist licenced for use in humans, ivabradine is available as a nonselective HCN antagonist for the treatment of chronic angina and heart failure. Ivabradine does not cross the blood–brain barrier and is therefore devoid of central nervous system (CNS) side effects which plague many current neuropathic pain medications. In the same sciatic nerve injury and chemotherapy-induced neuropathic pain models, ivabradine was equianalgesic with gabapentin.^45^ We previously conducted a crossover randomized controlled trial examining the effect of ivabradine on capsaicin-induced pain in healthy human volunteers.^20^ There were no significant effects of the drug on spontaneous pain or hyperalgesia. However, only a single 15-mg dose of ivabradine was administered. In addition, capsaicin causes neurogenic inflammation acutely, and the mechanisms underlying chronic neuropathic may pain differ.^44^ Here, we report the effects of ivabradine administered for at least 6 weeks in patients with chronic peripheral neuropathic pain.

## 2. Methods

The study was conducted in accordance with the spirit and the letter of the Declaration of Helsinki, the conditions and principles of International Conference on Harmonization's Good Clinical Practice, and the protocol and applicable local regulatory requirements and laws. The study was registered prospectively (ISRCTN68734605), and ethical approval was obtained from the London-Bromley Research Ethics Committee (16/LO/1901). Written informed consent was obtained from every participant before any study-related activity was performed.

### 2.1. Study design

This was a single-centre, open-label, feasibility study to assess the effect of ivabradine on chronic peripheral neuropathic pain in humans. Preclinical data suggest a strong dose–response relationship for the effect of ivabradine on heart rate and pain.^45^ Maximal analgesia is achieved at higher doses, when the effect of ivabradine on heart rate plateaus in humans.^30,40^ However, the risk of cardiac side effects at high doses in patients with sensory or autonomic neuropathies is unknown; using a fixed maximum dose design may have led to adverse events and participant withdrawal. To avoid this, we adopted an open-label design in which dose could be increased incrementally, titrating to heart rate and subjective symptoms. We additionally included a washout period after dosing to increase the sensitivity of the exploratory analyses. This dose–response design optimises detection of analgesic effect while ensuring the participants' safety.

### 2.2. Participants

**Figure 1.**
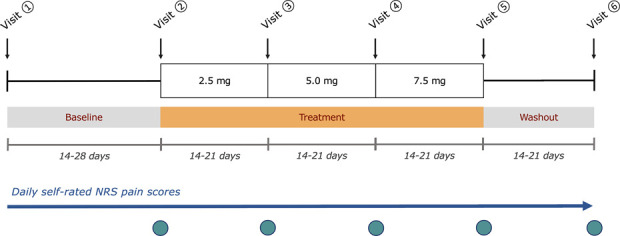
Schematic of trial design. This was a single-arm design in which all participants went through a schedule of baseline (Baseline), incrementally increasing dose (Treatment), and drug washout (Washout) periods. Participants rated their daily numerical rating scale (NRS) pain score in a pain diary. Secondary measures were recorded at visits 2 to 6, shown as teal circles.

Potential participants were identified from specialist clinics in Addenbrooke's Hospital and local general practices. We provided respondents with a participant information sheet and invited them for a screening visit at the Addenbrooke's Centre of Clinical Investigation in Cambridge. The following assessments were recorded as part of screening (visit 1, Fig. 1): clinical history, physical examination, medication use, and 12-lead electrocardiogram (ECG). We used Douleur Neuropathique 4 (DN4) and quantitative sensory testing (QST) to ascertain the neuropathic nature of pain and the 36-Item Short-Form Survey (SF-36) to assess overall health status.^4,42^

Participants had to have had a diagnosis of peripheral neuropathy with pain for over 6 months which scored more than 4 on the DN4, a scoring matrix for pain with neuropathic qualities in the history and on clinical examination. Participants with any significant cardiac comorbidity were excluded. The participant inclusion and exclusion criteria are shown in Table 1. All participants who met the eligibility criteria and gave written informed consent were included.

**Table 1:** Summary of inclusion and exclusion criteria for study participants.

Inclusion criteria	Exclusion criteria
Be able to give voluntary written consent	Known allergy to ivabradine; pre-existing treatment with ivabradine
Be aged 18 years or older	Use of drugs known to interact with ivabradine; use of prohibited concomitant analgesia; use of recreational drugs or excess alcohol
Have a diagnosis of peripheral neuropathy with pain, with pain for ≥6 mo and a DN4 score ≥4 and NRS score ≥4	Scheduled for any clinical treatment (surgical, pharmacological, interventional, and psychological) to begin during the trial
Be registered with a general practitioner	Be unwilling for the general practitioner to be notified
Have the following ECG characteristics: normal sinus rhythm, PR ≤ 210, QTcB ≤ 430 (men) ≤ 450 (women), QRS ≤ 120, heart rate ≥60 beats per minute	Significant cardiovascular comorbidity,* including heart failure, severe cardiac conduction abnormality, or postural hypotension
	Significant renal or hepatic impairment†
	Pregnancy, breastfeeding, or unwillingness to use contraception during the trial (both men and women)

* The full list of proscribed cardiovascular comorbidities can be found in the supplementary material, available at http://links.lww.com/PR9/A134.

† The full list of proscribed thresholds for renal and hepatic function can be found in the supplementary material, available at http://links.lww.com/PR9/A134.

NRS, numerical rating scale.

### 2.3. Dosing

The active treatment consisted of oral ivabradine, dosed at between 2.5 mg and 7.5 mg twice daily. The starting dose was 2.5 mg and was incrementally increased after a review of adverse effects and ECG monitoring at clinic visits every 2 to 3 weeks, to a maximum of 7.5 mg or a heart rate of 50 to 60 BPM (Fig. 1). This is a similar dosing schedule for the treatment of stable angina, which is started at 5 mg and increased to 7.5 mg twice daily.^13^

### 2.4. Study assessments

Participants reported pain on an 11-point numerical rating scale (NRS) (0 = no pain; 10 = worst possible pain) implemented on a short message service (FAST, Cambridge Digital Health). Participants were instructed and reminded to rate pain whose qualities are neuropathic in nature (as described by the adjectives in the DN4 questionnaire) and which was localised to the affected site. Daily text-based reminders were sent at a time of the participant's choosing. They were also allowed to send in ratings ad libitum. This was set up at screening and continued throughout the study.

At study visits 2 to 6, we administered the following questionnaires to capture the secondary outcome measures for chronic pain: brief pain inventory,^1,38^ Insomnia Severity Index (ISI),^2^ Pain Disability Index,^37^ and Depression, Anxiety, and Positive Outlook Scale.^26^ The Neuropathic Pain Symptom Inventory and QST on the affected area were used to assess the neuropathic characteristics of the pain.^5,32^ Quantitative sensory testing punctate pain threshold was taken as the mean of the weight at which pain is felt when a set of 7 standardized punctate probes (8–512 mN in weight) were applied in ascending and descending series. Quantitative sensory testing dynamic allodynia was measured as the perceived pain, rated from 0 to 100, when a standardized soft brush was applied as a single 2-cm stroke. For both measures, there was a minimum stimulus interval of 10 seconds.

### 2.5. Endpoints

The primary endpoint was the change from baseline in mean daily NRS pain score after dosing. The baseline mean was the mean of all daily NRS pain scores before dose initiation. The treatment mean was the mean of all daily NRS pain scores in the 10 days before ivabradine cessation at visit 5. This ignores entries in the first 4 days after dose adjustment, allowing ivabradine to reach steady state in the plasma.^34^ The secondary endpoints were the change from baseline in mean scores for each of the other measures listed in the previous section. The score at dose initiation was taken as baseline; the score at dose cessation (visit 5) was taken as treatment. Exploratory endpoints included examining the above measures for treatment vs washout, differences in baseline characteristics and individual participants' pain diagnoses, and using ECG heart rate measurements to explain variation in the primary and secondary endpoints.

### 2.6. Sample size calculation

We wanted to power for biologically significant or clinically useful effects that are unlikely to be explained by placebo-based analgesia. Placebo effects are well established, and Cochrane meta-analyses have estimated that the placebo-based reduction of pain scores is a standardized mean difference of −0.23 (95% CI −0.28 to −0.17).^14^ These analyses suggest that placebo-based analgesia would be expected to achieve a 1-point reduction on an 11-point NRS. Hence, we chose to power for a greater than 1-point NRS reduction; the null hypothesis was that the mean reduction in pain score is less than 1. Initial calculations suggested 36 participants would be required to correctly reject the null hypothesis at 80% power and a type 1 error rate of 5%.

### 2.7. Statistical analysis

We used OpenClinica (Community Edition) to capture data electronically and R Studio (Version 1.3.1093) to conduct statistical testing and generate graphs.^27,33^ Continuous outcome variables were summarized using the descriptive statistics n, mean, SD. The primary and secondary endpoints were analysed with 2-tailed dependent *t* tests comparing baseline mean with treatment mean, as per the protocol. To determine whether the effect of the drug on pain outlasts its presence in plasma, we ran 2-tailed dependent *t* tests comparing treatment mean with washout mean as exploratory analyses.

Participants provided at least 1 daily NRS pain score throughout the study period, producing a data set that was not fully used by the primary endpoint analysis. Using all available pain scores from each individual, we explored the effects of ivabradine dose and time on daily NRS pain scores in a linear mixed effects model.^3^ The fixed effects were dose and time; the random effect was participant level. Mixed effects models are well suited to longitudinal data because they take into account the correlated nature of repeated measures from the same subject and can account for multiple factors that may affect the measures differently over time. In addition, they can handle missing or imbalanced data, as is the case here where participants did not provide the same number of pain ratings per day.^8,25^

## 3. Results

The study was discontinued because of challenges to recruitment. Data from individuals who completed the study are presented. From a pooled general practice population of 90,000, we identified and invited 201 patients. Twenty-five subjects attended the screening visit; of those, 7 were eligible. These 7 completed the study schedule as described in a 17-month period beginning in July 2017. One participant (subject 24) did not attend a follow-up visit, but continued to submit NRS pain scores in the washout period so was still included. Baseline characteristics are shown in Table 2.

**Table 2:** Baseline characteristics of the participants receiving ivabradine for chronic peripheral neuropathic pain.

Subject	Sex	Age	BMI	ECG rate	Pain aetiology	DN4 total	SF-36 physical	SF-36 vitality	SF-36 mental health	SF-36 general health	Concomitant analgesia	Other medication
1	Male	55	27.6	113	Diabetic	4	10	15	56	10	N/A	Insulin SC 15 IU daily
2	Male	66	38.4	68	Diabetic	6	50	45	96	70	Naproxen 500 mg OD and duloxetine 30 mg OD	Metformin 500 mg BD, allopurinol 300 mg OD, atorvastatin 20 mg OD, and omeprazole 20 mg OD
6	Male	49	26.2	87	Amputation	7	0	40	60	70	Gabapentin 1200 mg TDS, amitriptyline 20 mg OD, and paracetamol 1 g PRN	
11	Male	66	21.8	71	Idiopathic	3	80	55	48	45	Gabapentin 900 mg TDS and Amitriptyline 10 mg OD	Cholecalciferol 800 mg OD
17	Female	44	33.1	90	Chemotherapy	8	20	20	84	50	Duloxetine 60 mg OD, paracetamol 1 g QDS, pregabalin 300 mg BD, and oramorph 10 mg 4 hrly	Omeprazole 20 mg BD, pyridoxine 50 mL TDS, tamoxifen 20 mg OD, zopiclone 3.75 mg OD, hypromellose 10 mcl PRN, and Hylo-Forte 1 drop QDS
21	Male	77	37.3	81	Idiopathic	8	60	40	72	50	Gabapentin 300 mg QDS and ibuprofen 400 mg OD	Allopurinol 400 mg OD, mometasone 50 mcg OD, and rabeprazole 20 mg BD
24	Male	64	25.6	71	Prolapsed disc	6	95	85	88	90	N/A	Montelukast 10 mg OD, Clenil Modulite 100 mcg BD, lansoprazole 15 mg OD, candesartan 8 mg OD, amlodipine 5 mg OD, salbutamol 100 mcg BD, and rosuvastatin 5 mg OD

BD, twice daily; BMI, body mass index; DN4, Douleur Neuropathique 4; ECG rate, electrocardiogram heart rate; IU, insulin units; OD, once daily; PRN, as required; QDS, four times daily; SC, subcutaneous; SF-36, Short-Form 36 Health Status Questionnaire; TDS, three times daily.

### 3.1. Effect of ivabradine on primary and secondary endpoints

Descriptive statistics for each outcome measure at baseline, at the end of treatment, and the washout period are shown in Table 3, top panel. There was no significant treatment effect on the primary endpoint, the change from baseline in mean NRS pain score with treatment: difference = −0.878, 95% CI = −2.07 to 0.31, *P* = 0.100 (Table 4). Post hoc sensitivity analysis using a power of 0.80 and alpha of 0.05 indicated that with a sample of 7, we were powered to detect a reduction in mean NRS of 2.20. There was a significant treatment effect on 1 secondary endpoint, the change from baseline in mean ISI score with treatment: difference = −0.457, 95% CI = −1.42 to −7.72, *P* = 0.012 (Table 4). Exploratory analysis of the difference between the end of treatment and washout scores for each primary and secondary outcome measure did not reveal any significant treatment effects (Table 4).

**Table 3:** Descriptive statistics for primary and secondary outcome measures at baseline, during treatment, and after washout.

Outcome	Baseline mean (SD)	Treatment mean (SD)	Washout mean (SD)
NRS, mean daily	5.39 (1.24)	4.52 (2.11)	5.48 (1.46)
BPI	5.11 (1.50)	4.21 (1.99)	5.21 (1.72)
ISI	12.29 (7.41)	7.71 (6.42)	8.43 (5.59)
NPSI	26.14 (13.84)	24.57 (19.61)	32.29 (18.84)
PDI	29.14 (20.98)	21.57 (23.50)	24.57 (21.48)
DAPOS depression	7.43 (2.37)	8.71 (4.07)	8.00 (2.52)
DAPOS anxiety	4.71 (2.14)	3.43 (0.53)	4.29 (1.50
DAPOS positive	8.86 (3.48)	8.00 (4.00)	8.00 (3.83)
QST-PPT	107.05 (132.03)	127.94 (134.04)	101.95 (124.07)
QST-DAS	6.54 (8.19)	3.91 (5.26)	10.91 (11.56)

Mean (SD).

BPI, brief pain inventory; DAPOS, Depression, Anxiety, and Positive Outlook Scale; DAS, Dynamic Allodynia Score; ISI, Insomnia Severity Index; NPSI, neuropathic pain symptom inventory; NRS, numerical rating scale pain score; PDI, Pain Disability Index; PPT, punctate pain threshold; QST, quantitative sensory testing.

**Table 4:** Two-tailed dependent *t* tests comparing the primary and secondary outcome measures for baseline and treatment, and treatment and washout, periods.

Outcome	Baseline vs treatment			Treatment vs washout		
	Difference	95% CI	*P*	Difference	95% CI	*P*
NRS, mean daily	−0.88	−2.07 to 0.31	0.100	0.97	−0.21 to 2.14	0.090
BPI	−0.89	−2.03 to 0.25	0.104	1.00	−0.33 to 2.33	0.115
ISI	−4.57	−1.42 to −7.72	0.012	0.71	−1.53 to 2.96	0.466
NPSI	−1.57	−14.70 to 11.56	0.780	7.71	−4.08 to 19.51	0.161
PDI	−7.57	−16.57 to 1.42	0.085	3.00	−28.44 to 34.44	0.823
DAPOS depression	1.29	−2.16 to 4.73	0.397	−0.71	−4.04 to 2.61	0.618
DAPOS anxiety	−1.29	NA	0.098	0.86	NA	0.203
DAPOS positive	−0.86	−2.50 to 0.78	0.248	0.00	−2.67 to 2.67	1.000
QST-PPT	20.89	NA	0.438	−25.99	NA	0.844
QST-DAS	−2.63	NA	0.181	7.00	NA	0.100

For outcomes in which the distributions were not normal (by the Shapiro–Wilk test), a Wilcoxon test was used, which does not report confidence intervals.

BPI, brief pain inventory; CI, confidence interval; DAPOS, Depression, Anxiety, and Positive Outlook Scale; DAS, Dynamic Allodynia Score; ISI, Insomnia Severity Index; NPSI, neuropathic pain symptom inventory; NRS, numerical rating scale pain score; PDI, Pain Disability Index; QST, quantitative sensory testing; PPT, punctate pain threshold.

### 3.2. Effect of ivabradine on heart rate

**Figure 2.**
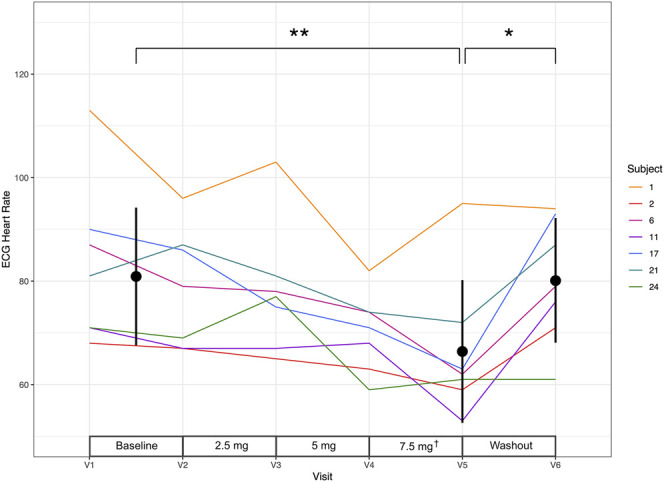
ECG-derived heart rate for each participant. The point ranges show mean and SD, for baseline, treatment, and washout. Baseline heart rate for each participant is the mean of both predosing ECG heart rate values ([V1 + V2]/2). The significance bars refer to *t* tests conducted on these mean values, when *<0.05 and **<0.01. Also shown is an indication of ivabradine dose between the study visits. ^†^Please note 1 participant, 24, failed to reach maximum dosing of 7.5 mg and hence received 5 mg until dose cessation.

Analysis of the effect of ivabradine on heart rate revealed a significant effect when comparing baseline and treatment (difference = −12.29, 95% CI = −5.62 to −18.95, *P* = 0.004), treatment and washout (difference = +13.71, 95% CI = 2.99 to 24.44, *P* = 0.020), and when using a linear regression model to assess the effect of dose on heart rate (F(1,39) = 10.4, *P* = 0.003, *R*^2^ = 0.21). Individual participant heart rate data are shown in Figure 2.

### 3.3. Effect of ivabradine on individual daily numerical rating scale pain scores

**Figure 3.**
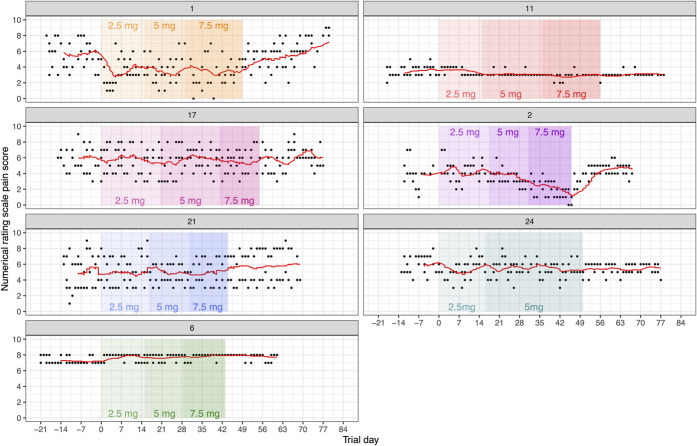
Daily numerical rating scale (NRS) pain score during the trial. Day 0 indicates the start of dosing. Each panel shows all the NRS scores provided by an individual. 7-day moving average for NRS score is shown in red. Increasing dose is represented by deepening rectangle colour, coloured by participant.

The raw daily NRS pain score data for each subject are shown in Figure 3, for interest. We conducted exploratory, hypothesis-generating analysis using a linear mixed effects model with dose and time as fixed effects and participant as a random effect; this allows the intercepts for putative linear relationships between dose, time, and NRS to vary by participant. This accounts for the difference in baseline NRS pain scores between participants. The analysis revealed a highly significant relationship between ivabradine dose and daily NRS pain scores (χ^2^(1) = 74.6, *P* < 0.001), with a reduction of 0.12 ± 0.01 (SEM) NRS points per milligram of ivabradine, and a significant relationship between time and daily NRS pain score ((χ^2^(1) = 74.6, *P* = 0.012), with an increase of 0.004 ± 0.002 (SEM) NRS points per trial day.

### 3.4. Adverse effects of ivabradine

The dosing schedule of ivabradine was very well tolerated in the participants. Only 1 participant reported dizziness on the 7.5-mg dose, which resolved spontaneously with no residual effects. None of the participants reported luminous visual phenomena, a recognised side effect of ivabradine.^40^

## 4. Discussion

The role of HCN2 ion channels in nociceptor firing is now well established. Preclinical work using pharmacological blockade and targeted genetic deletion has demonstrated they are required for inflammatory and neuropathic pain.^6,7,9,11,12,17,19,22,31,45^ Accordingly, peripherally restricted HCN2 blockers devoid of adverse effects on the heart and CNS would be highly desirable. Until these are developed, we have ivabradine, a nonselective, peripherally restricted HCN blocker that is already clinically available. Preclinical work has shown that ivabradine has analgesic properties in behavioural and electrophysiological studies of neuropathic pain.^9,45^ We sought to translate these findings to patients with a diagnosis of peripheral neuropathic pain.

The primary endpoint for the study was the difference in mean of NRS scores obtained 10 days before the final treatment visit compared with baseline mean. For this endpoint, there was no significant analgesic effect of ivabradine. However, we failed to achieve the sample size required because of challenges to recruitment in a single centre. This was despite recruitment from both the secondary care pain service and a primary care population of almost 100,000 patients. In addition, during the study, we made substantial amendments (approved by ethical, scientific, and safety review) to increase reimbursement for travel to recruit beyond the local area, broaden inclusion criteria to include idiopathic neuropathic pain, and relax the ECG QTcB exclusion criteria, based on safety data from our previous trial.^17^ These recruitment challenges eventually led us to discontinue the trial, and hence, we were unpowered for the primary endpoint. Post hoc sensitivity analysis suggested that with n = 7, we were only powered to detect an effect of 2.2 reduction in NRS pain score. Although we did not detect any significant analgesic effect of ivabradine, there was a dose-dependent reduction in participants' heart rate. This is unsurprising, given that the slowing of heart rate is an established drug effect.^13^ This finding usefully indicates that the participants adhered to the dosing schedule, excluding noncompliance as a cause for the negative study.

Data in mice suggest that the analgesic effect of ivabradine is also dose-dependent, with approximately the same ED50 for heart rate and analgesia at 2 to 2.5 mg/kg.^45^ This is consistent with ivabradine blocking nociceptor HCN2 channels and cardiac HCN4 channels approximately equally.^36^ Our previous human trial of ivabradine in acute capsaicin-induced pain also identified a robust dose-dependent reduction in heart rate, but no significant analgesic effect.^20^ That trial used a single dose in healthy participants under observation, so a dose of 15 mg was used, twice the upper dose used here. At approximately 0.2 mg/kg and 0.1 mg/kg, respectively, these are more than 10 times lower than the indicative analgesic ED50 in mice. In this study of peripheral neuropathic pain, incremental doses of ivabradine were used in periods that varied between 2 and 3 weeks between participants (Fig. 3). We were therefore able to explore ivabradine dose–response in a linear mixed effects model, by using all the pain ratings provided by the 7 participants during the study. We found a highly significant but modest effect of ivabradine dose on NRS pain score (0.12 NRS points reduction per milligram, *P* < 0.001), suggesting we would need to use at least 10 mg twice daily to see an effect above what would be expected with placebo analgesia. It is important to acknowledge that this finding is primarily driven by data from 2 participants (Fig. 3). Interestingly, although the study recruited from a range of aetiologies for peripheral neuropathic pain, we note that the 2 participants who responded are the 2 with painful diabetic neuropathy. In mouse models of both type I and type II diabetic neuropathy, high-dose ivabradine (5 mg/kg) had a profound effect on pain, reducing mechanical pain thresholds back to baseline (before the induction of diabetes).^39^ Diabetes is the leading cause of peripheral neuropathy worldwide, and when surveyed, between 13% and 34% of these patients report significant pain.^16^ It is important not to overinterpret the observations from just 2 patients, but this apparent analgesic effect in cases of painful diabetic neuropathy may merit further study.

The heterogeneity in neuropathic aetiology is 1 potential explanation for the negative result; we did not observe a large analgesic response in the patients with nondiabetic pain. However, although there may be particular benefit in diabetic neuropathy, the preclinical data demonstrate the role of HCN channels and analgesic effect of ivabradine across neuropathic aetiologies, including traumatic nerve lesions and chemotherapy-induced neuropathy. Another potential confounder is the variation in medication use at baseline. Participants taking any drugs on the British National Formulary's ivabradine interaction information were excluded; this includes inducers and inhibitors of cytochrome P450 3A4, the enzyme responsible for the metabolism of ivabradine, reducing the potential for pharmacokinetic interaction from heterogenous concomitant medications.^40^ The effect of concomitant analgesia is more difficult to quantify and control for. Two patients reported no concomitant analgesia. To reduce the effect of this, patients were required to keep their daily concomitant analgesia the same throughout the trial.

Acknowledging the limitations of the exploratory analysis and very small sample size, our findings suggest that a higher dose beyond 15 mg daily is required for clinically evident analgesic effect. In healthy humans, a 20-mg daily dose of ivabradine for 5 days causes a 17% to 20% reduction in heart rate, which is asymptomatic.^30^ There are also safety data suggesting a trend towards a plateau effect on heart rate at higher doses, with reduced risk of symptomatic bradycardia.^40^ In this feasibility study, for safety reasons, we titrated dose to heart rate up to the maximum of 15 mg daily as allowed in the licenced posology of the drug. This meant the trial was necessarily open label. Open-label studies are subject to a high risk of bias from both the participants and the clinicians. In addition, we acknowledge the lack of placebo or control arm. Hence, both group and individual results should be taken as exploratory in nature. Notably, there were no adverse effects. Therefore, we recommend that these exploratory results are further investigated in randomised, placebo-controlled trials. In that regard, placebo effects driven by positive expectations of benefit are highly pertinent in human trials and can be substantial in pain medicine.^21^ These effects seem to have increased over time in the United States.^41^ There are also data to suggest that open-label placebos, in which participants are placebo-aware, still have analgesic effects.^43^

Chronic neuropathic pain remains extremely difficult to manage and places a great burden on individuals, their families and carers, and society as a whole. Here, we report the first human trial of ivabradine for chronic pain. Exploratory analysis of our data suggests there may be an analgesic effect of ivabradine at higher doses, particularly in diabetic neuropathic pain. Our experience indicates that unlike current drugs for neuropathic pain, ivabradine is very well tolerated and devoid of CNS side effects. Furthermore, the drug is now off-patent. We believe that placebo-controlled studies are warranted to ascertain ivabradine's analgesic potential, investigating currently licenced doses in painful diabetic neuropathy, or higher doses in patients who have neuropathic pain but are otherwise healthy.

## Disclosures

The views expressed are those of the author(s) and not necessarily those of the NHS, the NIHR, or the Department of Health and Social Care. P. McNaughton is involved in a drug discovery programme in collaboration with Merck & Co, Inc, to develop HCN2-selective molecules as analgesics. The remaining authors have no conflicts of interests to declare.

## Appendix A. Supplemental digital content

Supplemental digital content associated with this article can be found online at http://links.lww.com/PR9/A134.
